# Diagnostic performance of multimodal ultrasound-based deep learning models in differentiating benign and malignant thyroid nodules

**DOI:** 10.3389/fonc.2026.1754676

**Published:** 2026-06-29

**Authors:** Huajie Ding, Lei Na, Meiling Hao, Wanlou Chen, Zhen Zhang

**Affiliations:** 1 Department of Ultrasound, Affiliated Hospital of Chengde Medical College, Chengde, China; 2 Department of Emergency, Affiliated Hospital of Chengde Medical College, Chengde, China; 3 Department of Pathology, Affiliated Hospital of Chengde Medical College, Chengde, China; 4 Department of Ultrasound, Liaoning Cancer Hospital & Institute, Shenyang, China

**Keywords:** convolutional neural networks, deep learning, diagnosis, thyroid nodule, ultrasonography

## Abstract

**Background:**

Multimodal ultrasound (US), including superb microvascular imaging (SMI) and shear-wave elastography (SWE), provides complementary information on tumor angiogenesis and tissue stiffness, which may enhance the diagnostic performance of deep learning (DL) models for thyroid nodules. However, current evidence remains limited and lacks comparative evaluation across different DL architectures. This study aimed to explore the performance of different DL models for differentiating benign and malignant thyroid nodules based on multimodal US images.

**Methods:**

This retrospective study involved 735 patients with surgically or pathologically confirmed thyroid nodules. A total of 15,373 multimodal US images, including B-mode (both longitudinal and transverse views), SMI, and SWE, were randomly divided at the image level into training (N = 11,530) and validation (N = 3,843) cohorts at a 3:1 ratio. Four convolutional neural networks, including ResNet50, DenseNet121, VGG16, and GoogLeNet, were trained and validated. The diagnostic performance of these models was compared with junior, intermediate, and senior radiologists. Gradient-weighted Class Activation Mapping (Grad-CAM) was employed to visualize the model’s areas of focus.

**Results:**

In the validation cohort, the ResNet50 model achieved the highest diagnostic performance [area under the curve (AUC): 0.931], followed by DenseNet121 (AUC = 0.857), VGG16 (AUC = 0.846), and GoogLeNet (AUC = 0.811). Delong’s test showed that the AUC of ResNet50 model was higher than that of the other models (all *P* < 0.001). Based on calibration analysis and the Hosmer-Lemeshow test, the overall calibration performance of the models was considered acceptable. The decision curve analysis suggested that ResNet50 provided the highest net clinical benefit. The diagnostic performance of the ResNet50 model (accuracy: 0.871) was numerically better than junior radiologists (accuracy: 0.810), comparable to intermediate radiologists (accuracy: 0.886), and lower than senior radiologists (accuracy: 0.946). Grad-CAM visualization suggested that the ResNet50 model mainly focused on clinically relevant thyroid nodule regions, with some misclassified cases showing excessive attention to local features.

**Conclusion:**

The multimodal US-based DL models achieve satisfactory performance in differentiating benign and malignant thyroid nodules, with the ResNet50 model possessing the highest performance, which may be comparable to intermediate radiologists.

## 1 Introduction

Thyroid cancer, one of the most common endocrine malignancies, accounts for over 0.8 million new cases in 2022 worldwide (1). Accurate differentiation between benign and malignant thyroid nodules is pivotal for avoiding unnecessary invasive procedures and delayed treatment (2). In clinical practice, multimodal ultrasound (US) is a routinely used method for thyroid nodule assessment, which integrates techniques like superb microvascular imaging (SMI) and shear-wave elastography (SWE) to offer comprehensive imaging data (3–5). However, the interpretation of multimodal US imaging is hampered by inherent subjectivity, inter-observer variability, and a strong dependence on radiologists’ experience.

Deep learning (DL), particularly convolutional neural networks (CNNs), achieves satisfactory performance in the classification of thyroid nodules (6–8). Several studies have demonstrated that DL models can achieve satisfactory diagnostic performance in distinguishing benign and malignant thyroid nodules based on US images (9–12). For example, a previous study reported that the US-based DL model achieved an average area under the curve (AUC) of 0.997 and an accuracy (ACC) of 0.984 in diagnosing benign and malignant thyroid nodules (9). Another previous study indicated that the US-based DL model achieved an overall diagnostic ACC of 73% in differentiating benign and malignant thyroid nodules, which was higher than that of 62.9% in the panel of radiologists (11). However, most existing models relied solely on single-mode US data (9–12), which mainly capture morphological features and may be insufficient for characterizing tumor biological behavior. In contrast, multimodal US techniques, including SMI and SWE, provide complementary information on tumor angiogenesis and tissue stiffness. Integrating structural and functional features through DL models may improve lesion characteristic learning and model’s robustness, thereby addressing the clinical need for more accurate risk stratification and fewer unnecessary invasive procedures. Although some previous studies explored multimodal US-based DL approaches, they mainly focused on the performance of a single CNN architecture without comparison across different models (13, 14).

Therefore, this study aimed to explore the performance of four DL models, including ResNet50, GoogLeNet, DenseNet121, and VGG16, for differentiating benign and malignant thyroid nodules based on multimodal US images.

## 2 Materials and methods

### 2.1 Patients

This study retrospectively collected 735 samples from 735 patients who underwent thyroidectomy or aspiration biopsy in the Department of Thyroid Surgery of our hospital between January 2022 and December 2023. The study was approved by the Ethics Committee of the Affiliated Hospital of Chengde Medical University (Approval No. CYFYLL2023313), with a waiver of informed consent.

The inclusion criteria were (1): preoperative thyroid nodule US examination completed (2); availability of both clinical US data and corresponding pathological diagnosis. The exclusion criteria were as follows (1): absence of pathological diagnosis (2); received I^131^ therapy or interventional ablation treatments before US examination (3); incomplete imaging due to excessively large nodules (4); poor imaging quality caused by nodule location or technical factors.

### 2.2 US data acquisition and pathological diagnosis

US examinations were performed using a GE LOGIQ E11 US system (GE Healthcare, USA) equipped with a 4–20 MHz adjustable wideband linear array transducer, supporting SMI and SWE. All examinations were performed in strict accordance with the manufacturer’s instructions. Patients were examined in the supine position with regular breathing, neck extended to expose the anterior cervical region. Longitudinal and transverse scans were obtained, and two representative images of each target nodule in the largest longitudinal and transverse planes were stored in DICOM format.

Image quality was independently reviewed by three US radiologists (junior: >2 and ≤8 years, intermediate: >8 and ≤15 years, senior: >15 years of experience) in thyroid nodule diagnosis, in accordance with the inclusion and exclusion criteria. Clinical and imaging information, including age, sex, nodule size, shape, echogenicity, margin, aspect ratio, composition, foci of strong echogenicity, multifocality, and blood flow grading (assessed by SMI), was recorded. Besides, pathological specimens were reviewed by three experienced pathologists. In cases of disagreement, a consensus diagnosis was reached through joint discussion.

### 2.3 Image preprocessing

All original US images were converted from DICOM to JPG format using MATLAB software to facilitate subsequent processing and training. The dataset included multimodal ultrasound images, including B-mode (longitudinal and transverse views), SMI, and SWE. Patient identifiers were removed. Prior to model development, all images were resized to 224 × 224 pixels and normalized to standardize pixel intensity values. The regions containing nodules were cropped to reduce background interference while preserving surrounding tissue structures. In addition, data augmentation techniques, including random rotation, horizontal and vertical flipping, scaling, and brightness adjustment, were applied to the training dataset to improve model robustness and reduce overfitting. Labels were assigned to each image according to pathological diagnoses, serving as the ground truth for benign versus malignant classification.

### 2.4 Development of DL diagnostic models

A dataset comprising 15,373 US images (including B-mode, SMI, and SWE) from 735 nodules was randomly divided at the image level into training and validation cohorts at a 3:1 ratio. The training set included 6,133 malignant and 5,397 benign nodules, while the validation set included 2,044 malignant and 1,799 benign nodules. All input images were standardized to a resolution of 224 × 224 pixels.

Four CNN architectures, ResNet50, GoogLeNet, DenseNet121, and VGG16, were employed to construct classification models. Pretrained weights were used as initialization. Model training was conducted on a Windows 10 (64-bit) operating system using Python and an NVIDIA GeForce RTX 2080Ti GPU. Hyperparameter tuning, including selection of the most suitable optimizer, was performed to improve training performance. The Adam optimizer was ultimately adopted to ensure model stability. Weight initialization was carefully considered to optimize convergence.

### 2.5 US physician diagnosis

Three US radiologists with different levels of experience independently read the images according to the Chinese Thyroid Imaging Reporting and Data System (C-TIRADS, 2020) (15), and SMI and SWE findings. Nodules categorized as C-TIRADS 1 to 3 were defined as benign. Those classified categorized as 4 and 5 were considered malignant. Of note, SMI was used as an additional imaging feature to assess vascularity. Blood flow signals were classified into four types according to vascular distribution patterns within thyroid nodules: type I (avascularity), type II (peripheral vascularity), type III (mainly peripheral vascularity), and type IV (mainly central vascularity). Types I-II were grouped as low vascularity and types III-IV as high vascularity. Regarding SWE, a semi-quantitative evaluation was performed based on color-coded elastography maps (red, yellow, green, and blue), where tissue stiffness was visually assessed. Clinically, nodules with predominantly blue regions were considered to have increased stiffness. No quantitative SWE parameters were used. To ensure diagnostic independence, no additional clinical or pathological information was provided to the radiologists.

### 2.6 US image visualization

To visualize the features learned by the DL models, Gradient-weighted Class Activation Mapping (Grad-CAM) was applied. During classification, the final fully connected layer integrates convolutional feature maps to generate class probabilities via a softmax function, with the maximum probability corresponding to the predicted category. Grad-CAM was used to compute a weighted linear combination of feature maps, followed by ReLU activation to produce heatmaps. In the visual outputs, critical feature regions were highlighted in warm colors (yellow), while less informative regions were indicated in cool colors (purple).

### 2.7 Statistical analysis

All statistical analyses were performed using SPSS version 23.0 and Python 3.7. Continuous variables were expressed as mean ± standard deviation (SD) and compared using Student’s t-test. Categorical variables were compared using the chi-square test. To evaluate the consistency of physicians’ assessments, inter-observer agreement was analyzed using Cohen’s kappa statistics. A *P* value < 0.05 was considered statistically significant. Model diagnostic performance was assessed using receiver operating characteristic (ROC) analysis, including the AUC, sensitivity (SEN), specificity (SPE), ACC, precision (PRE), and F1-score. Differences in AUCs between models were compared using the DeLong test. Model calibration was assessed using calibration curves and the Hosmer-Lemeshow test. To further assess the potential clinical utility of the models, decision curve analysis was conducted by calculating the standard net benefit across a range of threshold probabilities.

## 3 Results

### 3.1 Study workflow

**Figure 1:**
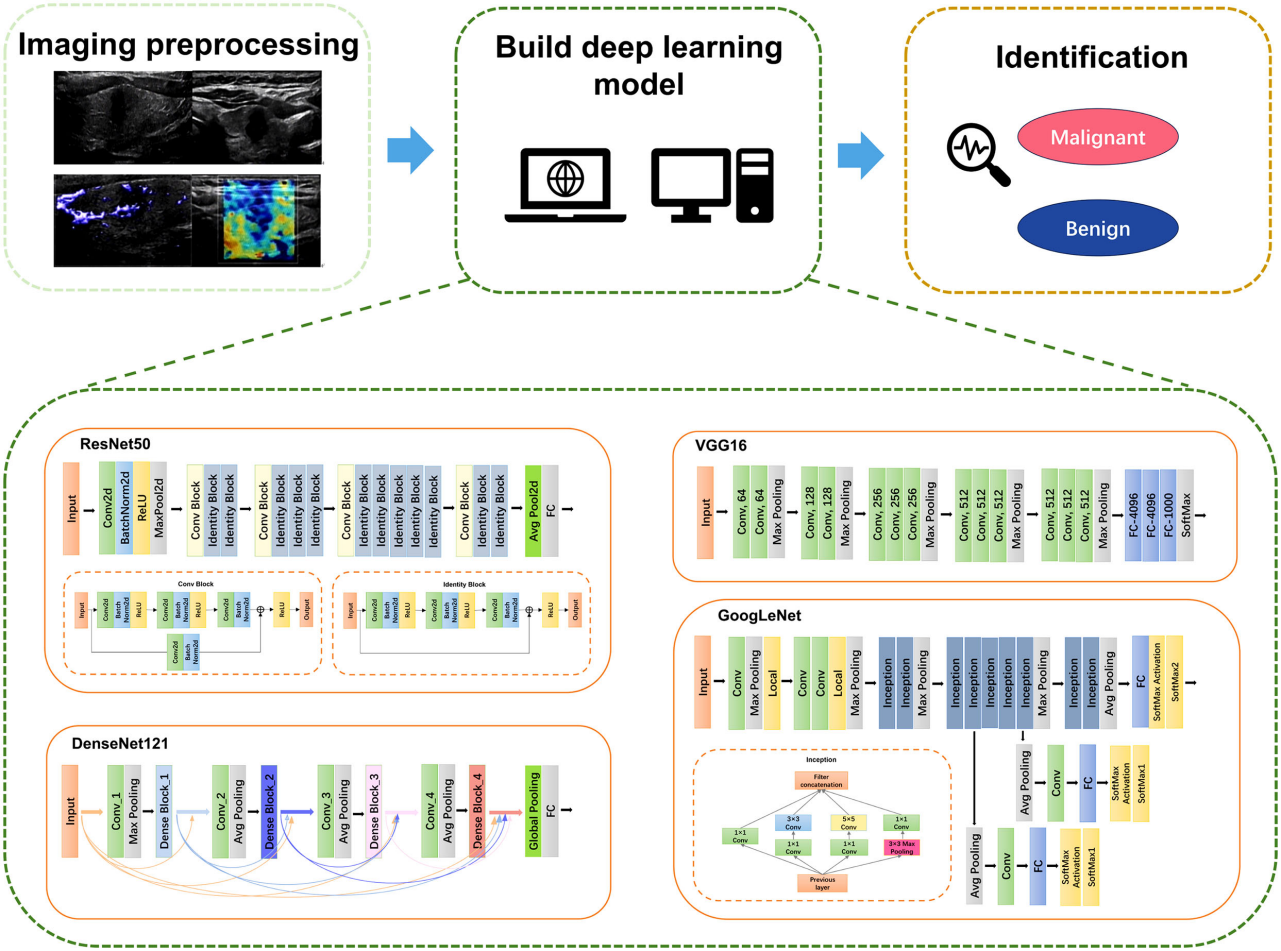
Schematic diagram.

The schematic diagram illustrated the sequential workflow for thyroid nodule diagnosis, encompassing image preprocessing, construction of four DL models with distinct architectures (ResNet50, DenseNet121, VGG16, and GoogLeNet), and final identification of thyroid nodules as malignant or benign (**Figure 1**).

### 3.2 Clinical and US features

In the training cohort, age, shape, nodule size, echogenicity, margin, aspect ratio, composition, foci of strong echogenicity, and multifocality differed between the malignant and benign groups (all *P* < 0.001). However, sex and blood flow grading (SMI) were not different between the two groups (both *P*>0.05) (**Table 1**).

**Table 1:** Clinical characteristics and ultrasound features.

Items	Training cohort (N = 551)			Validation cohort (N = 184)		
	Malignant (n = 292)	Benign (n = 259)	*P* value	Malignant (n = 97)	Benign (n = 87)	*P* value
Age (years), mean±SD	47.5±10.9	53.8±11.8	<0.001	45.5±10.0	50.3±11.8	0.024
Sex, n (%)			0.445			0.727
Female	244 (83.6)	210 (81.1)		30 (30.9)	29 (33.3)	
Male	48 (16.4)	49 (18.9)		67 (69.1)	58 (66.7)	
Shape, n (%)			<0.001			<0.001
Regular	38 (13.0)	238 (91.9)		11 (11.3)	78 (89.7)	
Irregular	254 (87.0)	21 (8.1)		86 (88.7)	9 (10.3)	
Nodule size, n (%)			<0.001			0.016
<1 cm	228 (78.1)	151 (58.3)		71 (73.2)	49 (56.3)	
≥1 cm	64 (21.9)	108 (41.7)		26 (26.8)	38 (43.7)	
Echogenicity, n (%)			<0.001			<0.001
Hypoechoic/Anechoic	274 (93.8)	57 (22.0)		85 (87.6)	17 (19.5)	
Isoechoic/Hyperechoic/mix	18 (6.2)	202 (78.0)		12 (12.4)	70 (80.5)	
Margin						
Boundary clarity, n (%)			<0.001			<0.001
Well-defined	51 (17.5)	237 (91.5)		15 (15.5)	79 (90.8)	
Poorly-defined	241 (82.5)	22 (8.5)		82 (84.5)	8 (9.2)	
Boundary with capsular, n (%)			<0.001			0.003
Clear	201 (68.8)	224 (86.5)		72 (74.2)	79 (90.8)	
Unclear	91 (31.2)	35 (13.5)		25 (25.8)	8 (9.2)	
Aspect ratio (A/T), n (%)			<0.001			<0.001
≤1	92 (31.5)	242 (93.4)		70 (72.2)	86 (98.9)	
>1	200 (68.5)	17 (6.6)		27 (27.8)	1 (1.1)	
Composition, n (%)			<0.001			<0.001
Solid	290 (99.3)	93 (35.9)		88 (90.7)	30 (34.5)	
Mixed cystic and solid	2 (0.7)	166 (64.1)		9 (9.3)	57 (65.5)	
Foci of strong echogenicity, n (%)			<0.001			<0.001
No	214 (73.3)	250 (96.5)		70 (72.2)	86 (98.9)	
Yes	78 (26.7)	9 (3.5)		27 (27.8)	1 (1.1)	
Multifocality, n (%)			<0.001			0.008
No	221 (75.7)	51 (19.7)		44 (45.4)	24 (27.6)	
Yes	71 (24.3)	208 (80.3)		53 (54.6)	63 (72.4)	
Blood flow grading (SMI), n (%)			0.112			0.042
I/II	173 (59.2)	136 (52.5)		49 (50.5)	31 (35.6)	
III/IV	119 (40.8)	123 (47.5)		48 (49.5)	56 (64.4)	

SD, standard deviation; A/T, anteroposterior diameter/transverse diameter; SMI, superb microvascular imaging.

In the validation cohort, age, shape, nodule size, echogenicity, margin, aspect ratio, composition, foci of strong echogenicity, multifocality, and blood flow grading (SMI) differed between the malignant and benign groups (all *P* < 0.05). Nevertheless, sex was not different between the two groups (*P* = 0.727) (**Table 1**).

### 3.3 Diagnostic performance of four models

In the training cohort, all four models exhibited an excellent ability to distinguish benign and malignant thyroid nodules. In detail, ResNet50, DenseNet121, VGG16, and GoogLeNet achieved AUCs of 0.997, 0.947, 0.998, and 0.995, respectively. Their corresponding ACCs were 0.976, 0.875, 0.978, and 0.970, respectively. The four models achieved SENs of 0.981, 0.914, 0.980, and 0.980, while SPEs were 0.970, 0.830, 0.975, and 0.959, respectively. Additionally, PREs were 0.974, 0.859, 0.978, and 0.964, and the F1 scores were 0.977, 0.886, 0.979, and 0.972, respectively (**Table 2**).

**Table 2:** Diagnostic efficiency of four different models.

Model	Training cohort						Validation cohort					
	AUC	ACC	SEN	SPE	PRE	F1 score	AUC	ACC	SEN	SPE	PRE	F1 score
ResNet50	0.997	0.976	0.981	0.970	0.974	0.977	0.931	0.871	0.862	0.882	0.892	0.876
DenseNet121	0.947	0.875	0.914	0.830	0.859	0.886	0.857	0.775	0.808	0.738	0.778	0.792
VGG16	0.998	0.978	0.980	0.975	0.978	0.979	0.846	0.763	0.885	0.626	0.728	0.799
GoogLeNet	0.995	0.970	0.980	0.959	0.964	0.972	0.811	0.733	0.736	0.731	0.756	0.746

AUC, area under the curve; ACC, accuracy; SEN, sensitivity; SPE, specificity; PRE, precision.

**Figure 2:**
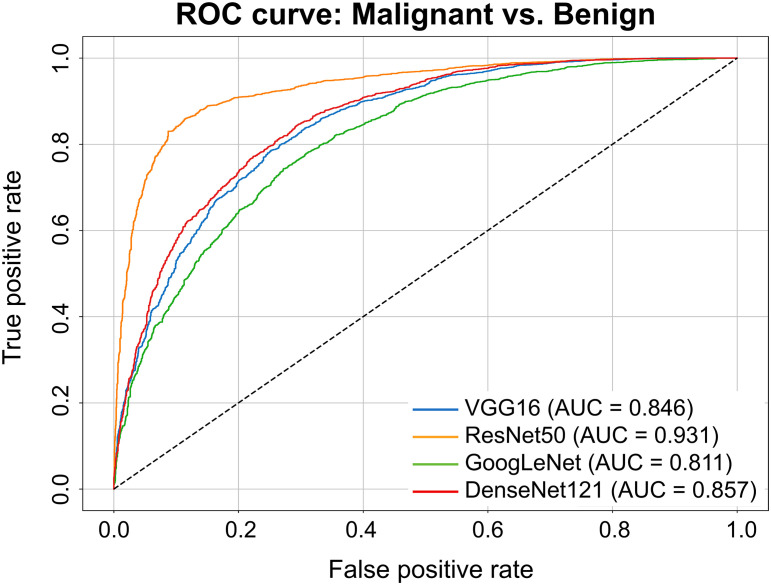
Models’ ability to distinguish between malignant and benign thyroid nodules.

In the validation cohort, ResNet50 demonstrated the best diagnostic performance with an AUC of 0.931, followed by DenseNet121 (AUC = 0.857) and VGG16 (AUC = 0.846), while GoogLeNet showed the lowest performance (AUC = 0.811) (**Figure 2**). Consistently, across other metrics (ACC, SEN, SPE, PRE, and F1-score), ResNet50 also achieved the highest values (0.871, 0.862, 0.882, 0.892, and 0.876, respectively), outperforming DenseNet121 (0.775, 0.808, 0.738, 0.778, 0.792), VGG16 (0.763, 0.885, 0.626, 0.728, 0.799), and GoogLeNet (0.733, 0.736, 0.731, 0.756, 0.746) (**Table 2**). The detailed information on the 95% confidence intervals (CIs) for AUC, SEN, and SPE ofeach model is shown in **Supplementary Table 1**.

### 3.4 Comparison, calibration, and clinical utility analyses of the four models

To compare the diagnostic performance of the four models, DeLong’s test was performed. The AUCs of the four models significantly differed (all *P* < 0.05). Of note, the AUC of ResNet50 was significantly higher than the other three models (all *P* < 0.001), suggesting its highest diagnostic performance (**Supplementary Table 2**).

Calibration curves demonstrated good agreement between predicted probabilities and observed outcomes for all four models, with DenseNet121 (mean absolute error: 0.008) and ResNet50 (mean absolute error: 0.010) exhibiting the closest alignment to the diagonal (**Supplementary Figure 1**). However, the Hosmer-Lemeshow test showed significant differences for all four models (all *P* < 0.001) (**Supplementary Table 3)**, suggesting discrepancies between predicted and observed probabilities. This finding may be attributable to the large sample size, as the Hosmer-Lemeshow test was sensitive in large datasets. Taking both calibration curves and Hosmer-Lemeshow test into account, the overall calibration performance of the models was considered acceptable.

Regarding clinical utility, decision curve analysis suggested that ResNet50 achieved the highest net benefit compared with the other three models, indicating the greatest potential clinical utility value (**Supplementary Figure 2**).

### 3.5 Diagnostic performance of radiologists

In the training cohort, the ACC, SEN, and SPE for distinguishing benign and malignant thyroid nodules were 0.816, 0.787, and 0.849 for junior radiologists; 0.884, 0.907, and 0.857 for intermediate radiologists; and 0.929, 0.921, and 0.938 for senior radiologists (**Table 3**).

**Table 3:** The diagnostic efficiency of junior, intermediate, and senior radiologists.

Items	Training cohort			Validation cohort		
	ACC	SEN	SPE	ACC	SEN	SPE
Junior	0.816	0.787	0.849	0.810	0.784	0.839
Intermediate	0.884	0.907	0.857	0.886	0.887	0.885
Senior	0.929	0.921	0.938	0.946	0.938	0.954

ACC, accuracy; SEN, sensitivity; SPE, specificity.

In the validation cohort, senior radiologists achieved the best diagnostic performance (ACC: 0.946, SEN: 0.938, SPE: 0.954), followed by intermediate radiologists (ACC: 0.886, SEN: 0.887, SPE: 0.885) and junior radiologists (ACC: 0.810, SEN: 0.784, SPE: 0.839) (**Table 3**). Compared with these radiologists, the diagnostic performance of the top-performing ResNet50 model (ACC: 0.871, SEN: 0.862, SPE: 0.882) was numerically better than junior radiologists, comparable to intermediate radiologists, and slightly lower than senior radiologists.

### 3.6 Inter-observer agreement among radiologists

The agreement between junior vs. intermediate radiologists (Kappa value: 0.58) and junior vs. senior (Kappa value: 0.64) was moderate. Substantial agreement was observed between intermediate vs. senior radiologists (Kappa value: 0.87) (**Supplementary Table 4**).

### 3.7 Image visualization analysis

**Figure 3:**
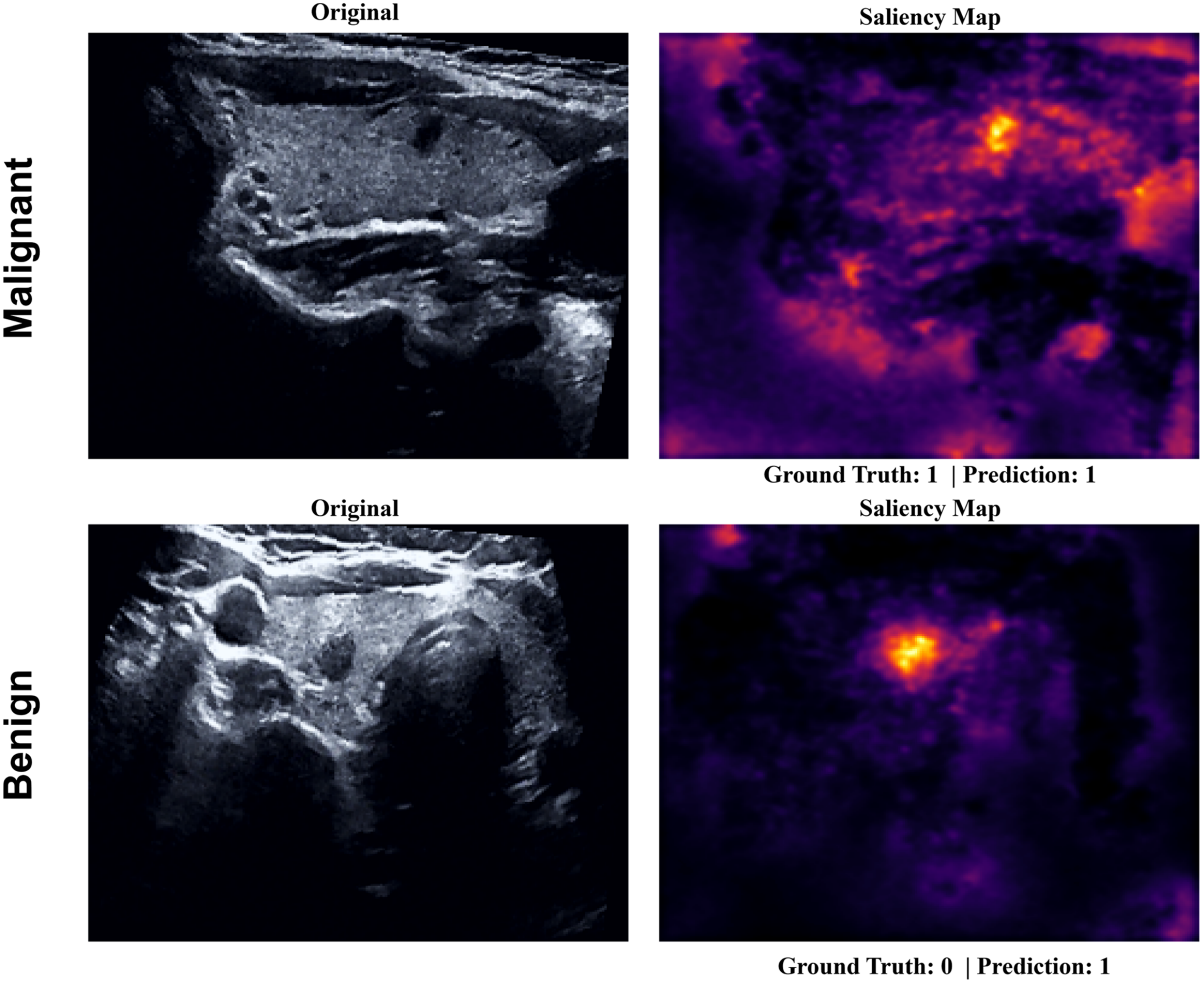
ResNet50 model interpretability using Grad-CAM.

To explore the underlying mechanism of ResNet50 in thyroid nodule classification, we used Grad-CAM to generate saliency heatmaps for visual interpretability analysis. As shown, the model assigned the highest attention weights (highlighted by yellow) to the thyroid nodule areas in both cases. In the malignant case, where the prediction matched the ground truth, the heatmap showed the model focused on the nodule and its adjacent tissue regions. In the benign case, where the model incorrectly predicted the nodule as malignant (prediction: 1 vs. ground truth: 0), the heatmap revealed that the model’s high attention was concentrated on a local region within the benign nodule, indicating that the misclassification might be driven by the model’s over-focus on atypical local features rather than the overall benign characteristics (**Figure 3**).

## 4 Discussion

DL models have demonstrated good performance in differentiating between benign and malignant thyroid nodules, owing to their sophisticated feature learning hierarchies (16–18). In this study, we developed and validated four DL models, including ResNet50, DenseNet121, VGG16, and GoogLeNet, for differentiating benign and malignant thyroid nodules using multimodal US images. These four models were selected due to their favorable performance in medical image analysis and their different architectural characteristics for feature extraction (19–21), which may help to better understand which type of DL architecture is more suitable for thyroid nodule classification and may further provide evidence to support clinical decision-making in thyroid nodule assessment. All these four models achieved good ability to discriminate benign and malignant thyroid nodules based on multimodal US images, with all AUCs >0.800 in the validation cohort. The performance of these four multimodal US-based DL models in diagnosing thyroid nodules was comparable to that of previous studies (19, 20, 22–25). Nevertheless, we found that the ResNet50 model showed better diagnostic performance than the other three models, achieving an AUC of 0.931, ACC of 0.871, SEN of 0.862, SPE of 0.882, PRE of 0.892, and F1 score of 0.876 in the validation cohort. The better diagnostic performance of the ResNet50 model might be attributed to its residual architecture, which was effective at capturing and fusing the complementary data provided by multimodal US, thereby enhancing classification performance (26). Consequently, this model might be a powerful tool for supporting radiologists in the discrimination of benign and malignant thyroid nodules. In contrast, VGG16, DenseNet121, and GoogLeNet, and did not perform well in differentiating thyroid nodules. The potential reasons might be that (1): VGG16, characterized by its relatively simple and sequential convolutional structure, might have limited capability in addressing complex multimodal feature interactions (2). DenseNet121’s performance might be affected by potential feature redundancy when handling heterogeneous multimodal inputs (3). GoogLeNet used multi-scale Inception modules, which might exhibit different feature integration characteristics compared with residual architectures. These architectural differences might partially explain the inferior diagnostic performance of these three DL models observed in the validation cohort. It should be noted that the AUCs of the four models in the training cohort (0.947-0.997) were obviously higher than that in the validation cohort (0.811-0.931), which was likely due to overfitting on the training data, which reduced model’s ability to generalize to unseen data.

DL models exhibited superior or comparable performance to radiologists in diagnosing thyroid nodules, according to previous studies (10, 11, 27). Two previous studies indicated that the US-based DL model exhibited a better ability to discriminate between benign and malignant thyroid nodules than radiologists (AUC: 0.922 vs. 0.839) (ACC: 0.730 vs. 0.629) (11, 27). Moreover, a previous study discovered that US-based DL models (AUC: 0.69) had comparable performance to radiologists in diagnosing thyroid nodules (AUC: 0.63 to 0.66) (10). In the current study, we discovered that the diagnostic performance (reflected by ACC, SEN, and SPE) of the top-performing ResNet50 model was numerically better than junior radiologists, comparable to intermediate radiologists, and slightly lower than senior radiologists. These findings suggested the potential of the multimodal US-based ResNet50 model as a powerful tool to support radiologists with inadequate experience in diagnosing thyroid nodules.

While the multimodal US-based ResNet50 model demonstrated a strong capability in discriminating between benign and malignant thyroid nodules, its clinical adoption is hampered by the black-box nature, which obscures the diagnostic mechanism (28). To solve this problem, we utilized the Grad-CAM to provide visual interpretations of the ResNet50 model’s classification decisions, enabling a qualitative assessment of its reasoning process (29). Saliency maps demonstrated that the ResNet50 model prioritized the core regions of the thyroid nodules over extraneous background tissue. The overlap between the highlighted areas and the actual thyroid nodules’ core regions supported the clinical validity of the ResNet50 model. Among the multimodal components, we speculated that SMI and SWE were likely the most valuable contributors, as they could reflect vascular information and stiffness features that are closely associated with tumor malignancy. By integrating these features on the basis of conventional morphological assessment, the ability of the ResNet50 model to differentiate thyroid nodules would be enhanced. Notably, the ResNet50 model’s reasoning pattern was similar to that of radiologists, which was crucial in building radiologists’ trust in the model and enabling collaborative diagnosis.

Compared with single-mode US, multimodal US could provide more comprehensive information on tumor malignancy. However, we noted that a previous study reported a remarkably high ACC of 0.984 using a single-mode US-based DL model (9), which exceeded the highest ACC achieved by ResNet50 (0.871) in our study. This discrepancy may raise questions regarding the incremental value of multimodal integration. Nevertheless, it should be clarified that the previous study was based on a relatively small dataset with only 508 US images, whereas our study involved 15,373 multimodal US images, introducing greater data heterogeneity and complex feature interactions. Moreover, integrating multimodal information might increase modeling complexity, potentially limiting the diagnostic performance of models. Therefore, the lower ACC observed in our study might not necessarily indicate that the use of multimodal US was worthless; instead, it would reflect the differences in data characteristics and task complexity between the two studies.

Vision Transformer (ViT) is a transformer-based architecture that replaces convolution operations with patch-based self-attention mechanisms for global feature modeling (30–32). Previous studies reported ViT achieved AUCs of 0.866-0.872 for thyroid nodule classification (33, 34), which were numerically lower than ResNet50 in our study. The model architectures differ between ViT and ResNet50. Specifically, ViT relies on global self-attention mechanisms to capture long-range dependencies, whereas ResNet50 is based on convolutional operations that emphasize local feature extraction and hierarchical representation. These structural distinctions might lead to the difference in diagnostic performance between these two models. However, considering differences in dataset characteristics, direct cross-study comparisons might not fully reflect the relative diagnostic performance for thyroid nodule classification, and further studies are warranted to evaluate ViT and CNN-based models within the same dataset.

Recent advances in artificial intelligence (AI), such as ChatGPT, a prominent large language model, have demonstrated growing potential in several medical domains, including thyroidology (35–38). For example, some studies have showed that ChatGPT may support various clinical tasks, such as thyroid disease counseling and risk stratification of thyroid nodules (39, 40). However, current evidence remains largely limited to controlled settings and indicates that ChatGPT should be restricted to an assistive role in clinical applications (35–37). Notably, the integration of ChatGPT with DL models may represent an emerging “ChatGPT-empowered DL” paradigm, which has the potential to facilitate the application of DL-based methods in thyroid nodule diagnosis by reducing the complexity of machine learning workflows and improving their accessibility in clinical settings (38). Based on the findings of the present study, the multimodal US-based ResNet50 model showed the highest diagnostic performance in differentiating benign and malignant thyroid nodules. Thus, ResNet50 may be further integrated with ChatGPT, which could potentially enhance workflow efficiency, facilitate clinical interpretation, and improve the accessibility of DL-based diagnostic tools in thyroid nodule assessment.

There were several limitations in this study (1). The retrospective design might introduce selection bias (2). All US images were obtained from a US machine, and no external validation was performed, which might affect the generalizability of our findings (3). In the selection of 2D US images, we chose the largest cross-sectional view. However, this method might not capture all classic features of the thyroid nodule, which could consequently affect the model’s diagnostic performance (4). Future studies should include prospective multi-center validation to assess model’s generalizability across different populations and ultrasound platforms. In addition, integration of the DL model with established TI-RADS risk stratification systems may enhance clinical applicability. Moreover, real-time deployment studies are needed to evaluate the feasibility of DL models in routine clinical settings (5). In this study, C-TIRADS 3 was used as the threshold for differentiating benign and malignant thyroid nodules. However, C-TIRADS 4B is also widely regarded as an important threshold, and further studies could consider applying this threshold to differentiate benign and malignant thyroid nodules (6). Grad-CAM was only assessed qualitatively. As no pixel-level or region-level annotations were available, quantitative evaluation of localization performance (e.g., using IoU or Dice) was not applicable, which should be further explored (7). The high AUC values in the training set might suggest potential overfitting, which would be partly attributed to the image-level split between the training and validation cohorts, leading to data correlation. Further validation using patient-level splitting and external datasets is needed to confirm the robustness of the findings (8). There was a significant bias in the gender distribution between the training and validation cohorts, which might affect the robustness and generalizability of our findings. Therefore, the findings of this study should be interpreted with caution, and further validation using larger and more balanced datasets is warranted.

## 5 Conclusion

In conclusion, the multimodal US-based DL models exhibit satisfactory performance in differentiating benign and malignant thyroid nodules, with the ResNet50 model possessing the highest performance, which may be comparable to intermediate radiologists.

## Data Availability

The original contributions presented in the study are included in the article/**Supplementary Material**. Further inquiries can be directed to the corresponding author.

## Supplementary material

Supplementary Figure 1 Calibration curves for the four models.

Supplementary Figure 2 Decision curve analysis for the four models.

Supplementary Table 1 95 CIs for AUC, SEN, and SPE.

Supplementary Table 2 Delong’s test among different models in validation cohort.

Supplementary Table 3 Hosmer-Lemeshow test of different models in validation cohort.

Supplementary Table 4 Consistency of assessments among junior, intermediate, and senior radiologists.
