# Supplementary figures and images for: Diagnostic performance of multimodal ultrasound-based deep learning models in differentiating benign and malignant thyroid nodules
Source: Front Oncol. 2026 Jun 29;16:1754676. doi: 10.3389/fonc.2026.1754676 (PMC13357126; doi:10.3389/fonc.2026.1754676)

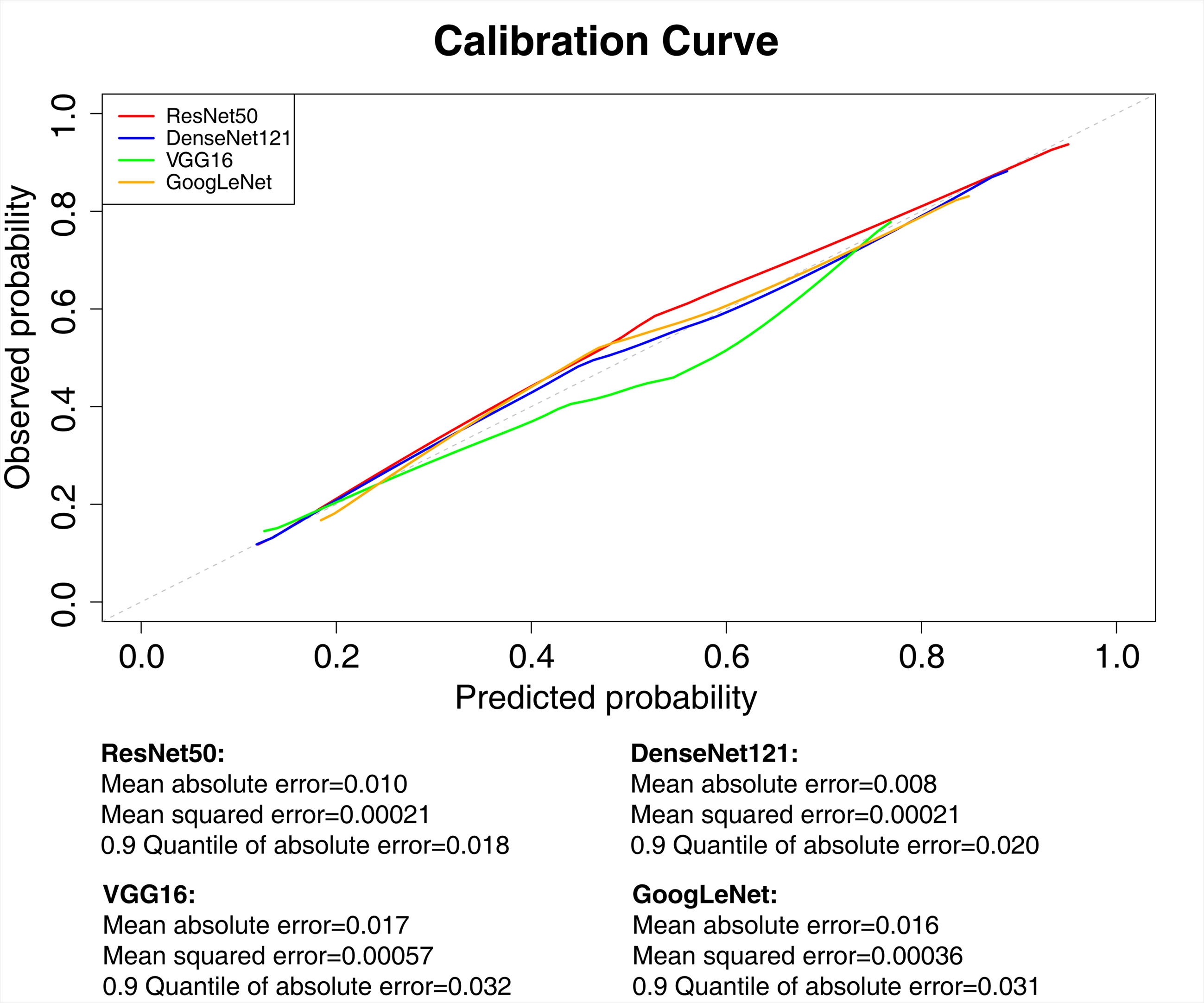

Supplement: Supplementary Figure 1 — Calibration curves for the four models. [file Image1.tif]

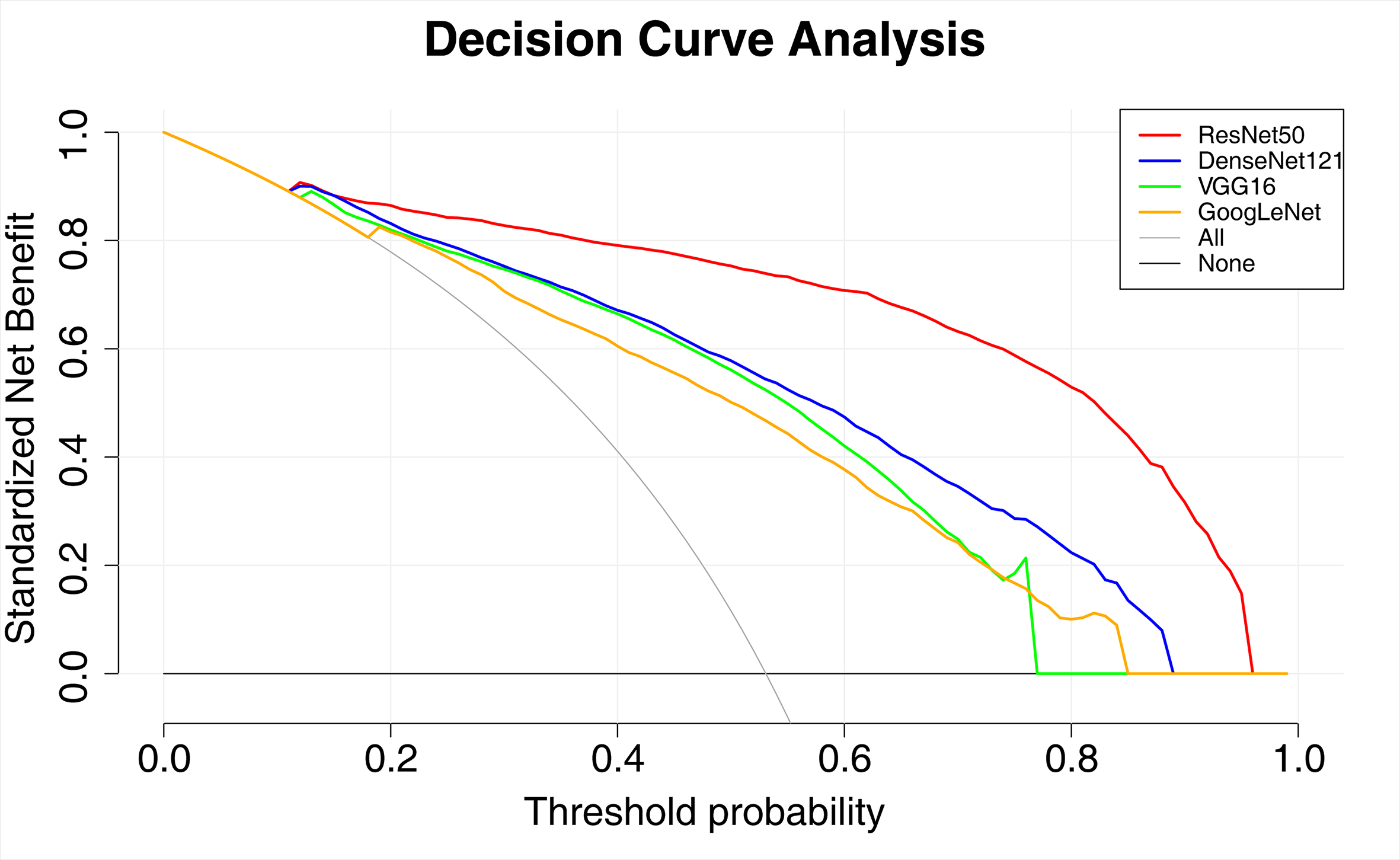

Supplement: Supplementary Figure 2 — Decision curve analysis for the four models. [file Image2.tif]
