# Supplementary material for: Diagnostic performance of multimodal ultrasound-based deep learning models in differentiating benign and malignant thyroid nodules
Source: Front Oncol. 2026 Jun 29;16:1754676. doi: 10.3389/fonc.2026.1754676 (PMC13357126; doi:10.3389/fonc.2026.1754676)
Supplement: Supplementary Table 1 — 95 CIs for AUC, SEN, and SPE. [file Table1.docx]

**Supplementary Table 1.** 95 CIs for AUC, SEN, and SPE.

| Model | Training cohort | | | Validation cohort | | |
| --- | --- | --- | --- | --- | --- | --- |
|  | AUC (95% CI) | SEN (95% CI) | SPE (95% CI) | AUC (95% CI) | SEN (95% CI) | SPE (95% CI) |
| ResNet50 | 0.997 (0.997-0.998) | 0.981 (0.977-0.984) | 0.970 (0.965-0.975) | 0.931 (0.924-0.939) | 0.862 (0.847-0.875) | 0.882 (0.867-0.895) |
| DenseNet121 | 0.947 (0.943-0.951) | 0.914 (0.907-0.922) | 0.830 (0.819-0.840) | 0.857 (0.846-0.868) | 0.808 (0.791-0.823) | 0.738 (0.719-0.757) |
| VGG16 | 0.998 (0.997-0.998) | 0.980 (0.976-0.984) | 0.975 (0.970-0.979) | 0.846 (0.835-0.857) | 0.885 (0.871-0.897) | 0.626 (0.605-0.647) |
| GoogLeNet | 0.995 (0.995-0.996) | 0.981 (0.977-0.984) | 0.959 (0.953-0.964) | 0.811 (0.799-0.824) | 0.736 (0.718-0.754) | 0.731 (0.712-0.750) |

AUC, area under the curve; CI, confidence interval; SEN, sensitivity; SPE, specificity.
