# Supplementary material for: Diagnostic performance of multimodal ultrasound-based deep learning models in differentiating benign and malignant thyroid nodules
Source: Front Oncol. 2026 Jun 29;16:1754676. doi: 10.3389/fonc.2026.1754676 (PMC13357126; doi:10.3389/fonc.2026.1754676)
Supplement: Supplementary Table 2 — Delong’s test among different models in validation cohort. [file Table2.docx]

**Supplementary Table 2.** Delong’s test among different models in validation cohort.

| Model | ResNet50 | DenseNet121 | VGG16 | GoogLeNet |
| --- | --- | --- | --- | --- |
| ResNet50 | (-) | Z = 11.167,  *P* <0.001 | Z = 12.540,  *P* <0.001 | Z = 16.371,  *P* <0.001 |
| DenseNet121 | Z = 11.167,  *P* <0.001 | (-) | Z = 2.018,  *P* = 0.044 | Z = 7.211,  *P* <0.001 |
| VGG16 | Z = 12.540,  *P* <0.001 | Z = 2.018,  *P* = 0.044 | (-) | Z = 5.311,  *P* <0.001 |
| GoogLeNet | Z = 16.371,  *P* <0.001 | Z = 7.211,  *P* <0.001 | Z = 5.311,  *P* <0.001 | (-) |
