# Supplementary material for: Diagnostic performance of multimodal ultrasound-based deep learning models in differentiating benign and malignant thyroid nodules
Source: Front Oncol. 2026 Jun 29;16:1754676. doi: 10.3389/fonc.2026.1754676 (PMC13357126; doi:10.3389/fonc.2026.1754676)
Supplement: Supplementary Table 3 — Hosmer-Lemeshow test of different models in validation cohort. [file Table3.docx]

**Supplementary Table 3.** Hosmer-Lemeshow test of different models in validation cohort.

| Model | Chi-square | *P* value | Number of groups |
| --- | --- | --- | --- |
| ResNet50 | 6568.300 | <0.001 | 10 |
| DenseNet121 | 882.850 | <0.001 | 10 |
| VGG16 | 118370.000 | <0.001 | 10 |
| GoogLeNet | 1884.300 | <0.001 | 10 |
