# Supplementary material for: Diagnostic performance of multimodal ultrasound-based deep learning models in differentiating benign and malignant thyroid nodules
Source: Front Oncol. 2026 Jun 29;16:1754676. doi: 10.3389/fonc.2026.1754676 (PMC13357126; doi:10.3389/fonc.2026.1754676)
Supplement: Supplementary Table 4 — Consistency of assessments among junior, intermediate, and senior radiologists. [file Table4.docx]

**Supplementary Table 4.** Consistency of assessments among junior, intermediate, and senior radiologists.

| Items | Kappa value |
| --- | --- |
| Junior vs. Intermediate | 0.58 |
| Junior vs. Senior | 0.64 |
| Intermediate vs. Senior | 0.87 |
